# Understanding Vanadium Ion Diffusion in Nafion Using an Atomistic Study and Microscopic Concentration Profiles

**DOI:** 10.3390/membranes16060195

**Published:** 2026-06-03

**Authors:** Sven Hampel, Christian Lutz, Gerald Falkenberg, Joanna Kolny-Olesiak, Ursula E. A. Fittschen, Nina Merkert

**Affiliations:** 1 Institute of Inorganic and Analytical Chemistry, Clausthal University of Technology, Arnold-Sommerfeld-Straße 4, 38678 Clausthal-Zellerfeld, Germany; sven.hampel@desy.de (S.H.); christian.lutz@alumni.tu-clausthal.de (C.L.); joanna.kolny.olesiak@tu-clausthal.de (J.K.-O.); ursula.fittschen@tu-clausthal.de (U.E.A.F.); 2 Deutsches Elektronen-Synchrotron DESY, Notkestraße 85, 22607 Hamburg, Germany; gerald.falkenberg@desy.de; 3 Institute of Metallurgy, Clausthal University of Technology, Arnold-Sommerfeld-Str. 6, 38678 Clausthal-Zellerfeld, Germany

**Keywords:** ionomeric membrane, molecular dynamics, XRF, unsteady diffusion

## Abstract

The functionality of ionomeric membranes is influenced by small changes of several parameters. Aqueous network formation by phase separation between the hydrophilic and hydrophobic parts of the polymer is one critical factor for water and ion transport. In particular, the transport of highly charged ions like $V^{3+}$ is not well understood. The unsteady diffusion in Nafion, a sulfonic acid based cation exchange polymer, using $V^{3+}$ profiles obtained with micro X-ray fluorescence (0.5 μm spot over a 180 μm scan) yields a diffusion coefficient of $4\times 10^{-13}\;m^{2}s^{-1}$ at $\lambda_{H_{2}O/SO_{3}}=12$ and at ca. 20 °C. It is confirmed that the concentration profile can be described by an error function formalism. The diffusivity, determined from the entire profile, represents mainly the transport into a vanadium free environment with very low ionic strength as the membrane was conditioned in ultra-pure water. The macroscopic ion transport is influenced by local molecular interactions, interconnection of water pockets and long range ionic interactions. The local interactions of $V^{3+}$ were studied using molecular dynamics (MDs) simulations. The MD simulation studies diffusion at a constant ion concentration and short length scale (ca. 30 nm). It gives insights on the effects of dissolved $V^{3+}$ ions on the local structure. Radial distribution functions reveal that at low hydration, the vanadium ions have an ordering effect on water molecules. The diffusion coefficient of $V^{3+}$ is determined on a molecular level from the mean-square displacement yielding $2.5\times 10^{-10}\;m^{2}s^{-1}$ for $V^{3+}$ ions at a membrane water content of $\lambda_{H_{2}O/SO_{3}}$ = 6. The phenomenon in which the diffusivity decreases over longer length scales was documented before for water and $H^{+}$ in Nafion; however, this was by only about one order of magnitude. The experimental microscopic approach described by us is universally applicable, e.g., to environments of higher ionic strength, ions with different charges, and different types of ion-exchange membranes. Longer diffusion times allow us to distinguish between different concentration regimes.

## 1. Introduction

Ionomeric membranes and especially perfluoro-sulfonic acid based cation exchange membranes [1] are widely applied in electrochemical systems. Their use has significantly improved operation from electrolysis cells [2], fuel cells [3] and redox flow batteries [4]. The transport of ions across the membrane is critical for the functionality of the devices. Selectivity of the transport is as important. Often, certain ion species need to be transported efficiently, e.g., protons for charge balance, while other ions should remain in their respective compartment. The efficiency of ion transport is highly sensitive to different parameters, the size and charge of the dissolved ion, of the ion-exchange group as well as the hydration level of the membrane. Higher temperatures will yield higher permeability. Sun et al. found a 3% rise in vanadium ion permeability in Nafion for each degree in temperature [5]. In general, high diffusion coefficients are found at high membrane water contents. The water content inside the membrane is decisive for the formation of an aqueous network and the mobility of ions. The aqueous network is established by phase separation between the hydrophilic and hydrophobic part of the polymer. The water content is usually determined by weight increase of the polymer after contact with water. It is given in terms of $\lambda_{H_{2}O/SO_{3}}$, which is the number of water molecules per ion-exchange group present in the polymer. Typical lambda values range from 2 to 20, where the latter is about the maximum hydration level in, i.e., Nafion 117. It depends on factors like ionic strength of the electrolyte [1]. Interaction of water molecules with the ion exchange groups as well as with dissolved ions are involved in membrane hydration. It was frequently reported that with increasing acid concentration the water content of the membrane decreases and subsequently the permeation flux [5,6,7]. However, according to experiments by Verbrugge and Hill, the porosity (correlated to $\lambda_{H_{2}O/SO_{3}}$) first increases for acid concentration in the reservoir from 0.0–0.1 M and then decreases [8]; the latter agrees with many other studies [5,6,7].

It is well known that the degree of hydration affects ion mobility inside the membrane. A combined modeling and experimental approach using Quasi Elastic Neutron Scattering (QENS) and Pulsed Field Gradient Nuclear Magnetic Resonance (PFG-NMR) to study diffusion of protons and water in perfluoro-sulfonic acid polymers (PFSA) identified for each probed length scale a different diffusion coefficient D. The authors attributed those to a hopping on the atomic scale, a local diffusion in water pouches and a transition in between those on the nanometer scale and self diffusion on the micro meter scale. The diffusion coefficient of water in Nafion decreased from short to longer scales, e.g., at a medium hydration level ($\lambda_{H_{2}O/SO_{3}}$ = 4.7) on the nanometer scale the D was about 0.7 × $10^{-9}\;m^{2}s^{-1}$ and on the micro meter scale only 0.08 × $10^{-9}\;m^{2}s^{-1}$ [9]. Their Molecular Dynamic (MD) simulation yielded over all higher Ds than their experiments. The diffusion of single charged ions, i.e., ${\mathrm{Na}}^{+}$ and ${\mathrm{Cl}}^{-}$ has been studied using MD simulations in a polyelectrolyte composed of the negatively charged poly(sodium-4-styrenesulfonate) (PSS) and the positively charged poly(diallyldimethylammonium chloride) (PDAC). The results also show a decrease in diffusivity with length and suggested a strong influence of electrostatic/steric trapping more complicated than the models on pocket trapping due to connect and disconnect inside the polymer water network [10]. Both studies derive anomalous diffusion from the mean-square displacement (MSD) at low hydration levels or short time scales, which deviates slightly from Equation (2), indicating a power-law [| $r^{2}$(t)$|\;\propto \;t^{n}$ with 0.6 < n < 1].

At low hydration levels ion interaction with water molecules is expected to be different from those at high hydration levels, the latter being close to bulk water. Their nature is subject to ongoing research and has been widely discussed in the literature. Inter-molecular attractions between high-valent ions, as the $V^{3+}$ studied here, with two or more ion-exchange groups have been suggested [1]. The interaction is discussed being either a solvent share of the free ions and the ion-exchange groups over a water molecule or a contact pairing of the ions. However, those are rather difficult to study. Infrared spectroscopy has been applied to distinguish between dissolved ions bound over a solvent-share mechanism with the polymer ion-exchange group, or over a direct ion pairing [11,12]. The experimental studies have not been conclusive on the mechanisms. MD simulations modeling only the Nafion side chain ($C_{5}F_{11}SO_{3}^{-}$) in a bulk water box with the different vanadium ions found, that only $V^{3+}$ showed preferences for a direct binding with the sulfonate group. This was in accordance with their gas phase DFT calculations [11]. MD simulations also have been used, e.g., to study the phase separation of anion exchange membranes in dependence of the hydrophilic side chain length [13].

MD simulations of hydrated Nafion usually focus on proton transfer and other singly charged ions as the ones discussed above [9,10,14,15,16]. Here, we study the dynamics of $V^{3+}$ inside lowly and highly hydrated Nafion 117. For comparison a vanadium ion free membrane is simulated. The obtained pair distribution functions give a measure of the degree of interaction of molecules inside the membranes at high and low hydration levels as well as with and without the highly charged $V^{3+}$. By evaluating the mean-square displacement of the ions, the diffusion coefficient on a short length scale (ca. 30 nm) is derived. Regarding the diffusion on the micro meter scale additional processes to the local interactions probed in the MD simulations play a crucial role, leading to sub-diffusion of water molecules as described above. Experimentally, the diffusivity of highly charged vanadium ions has been mainly determined over the entire length of the membrane, i.e., approx. 180 μm in macroscopic dialysis-cell approaches. The results deviate significantly, e.g., almost by a factor of 20 e.g., for $V^{3+}$ inside Nafion 117 and 115, from 7.12 × $10^{-13}\;m^{2}s^{-1}$ [7] to 1.45 × $10^{-11}\;m^{2}s^{-1}$ [17]. The dialysis cell approach is highly sensitive to additional potentials and the assumptions of the calibration constant. The latter is derived from the area open for diffusion, the thickness of the membrane and the volumes in the two compartments. An experimental approach better suited to probe short length scale phenomena in an unsteady state is developed here. The observed mobility of the $V^{3+}$ is the sum of the local diffusivity and other trapping and hopping mechanisms. In advantage over the dialysis cell approach, it is less sensitive to, e.g., Donnan potentials and the calibration constant. Here, the diffusion coefficient, as a measure of ion mobility including molecular interactions, is determined from a free diffusion experiment describing the unsteady diffusion in a semi-infinite slab. Here, particularly concentration profiles of $V^{3+}$ (after 600 s of diffusion time) obtained by micro X-ray fluorescence (micro-XRF) in hydrated Nafion were used for the determination of the diffusion coefficient. The membrane was plunge-frozen and cryo-cooled during the concentration profiling, to fixate the ion positions and prevent radiation damage. The rapid cooling prevents from ice crystal formation and ideally converts the water inside the membranes pore network to vitreous ice [18,19]. Crystal formation is expected to change the network structure and with that alter the ion distribution [1]. Previous experiments on cryo transmission electron microscopy of Nafion revealed the microstructure and showed that the process does not alter the morphology [20,21]. The set-up was miniaturized according to standards of the synchrotron beamline, which have proven successful for different biologic samples [22,23].

In previous work, we have determined the profiles of different vanadium species to study redox reactions inside the nanoscopic water body of hydrated Nafion by micro X-ray absorption near edge structure (XANES) using a similar experimental set-up as described in [24]. The diffusion coefficient D, for small concentration gradients, is in general, available by Fick’s second law for a one-dimensional approach [25]

(1) $$\frac{\partial c}{\partial t}=D\frac{\partial^{2}c}{\partial^{2}x}$$

where $\frac{\partial c}{\partial t}$ notes the time-dependent and $\frac{\partial^{2}c}{\partial^{2}x}$ the position dependent concentration gradient. The phenomena probed by this approach are in some aspects inherently different from those studied by steady-state experiments using permeability cells (most commonly applied in literature). Studying the vanadium profile has the potential to reveal differences in the “early”-ion mobility which diffuse into the vanadium-free membrane and the mobility of the ions that follow the front and diffuse into a now vanadium-filled environment. Though the magnitude of molecular interactions is not directly accessible, by conditioning the membrane with different ions it will be possible to study the influence of varying inner-membrane environments. In the work presented here, we establish a procedure to evaluate profiles from quite short diffusion times (600 s)—compared to the dialysis cell approaches—hence the diffusion into an initially vanadium ion free environment. Applying longer diffusion times, in the future it will be possible to study different diffusion regimes. Micro-XRF profiles have been applied previously in plant physiology research to study diffusion of $K^{+}$ and ${\mathrm{Cu}}^{2+}$ in wood cell wall layers by Jakes et al. [26], materials quite different from the PFSA membranes. Nonetheless, ion diffusion in polymer electrolyte membranes is of utmost importance as it affects the performance of all membrane based electrochemical cells. Laboratory based Micro-XRF has been used successfully studying vanadium ion diffusion *in-plane* of Nafion; however, it was not capable of offering the spatial resolution needed for *through-plane* determination [27]. The two approaches introduced here are complementary, in a way, where the MD simulation suggests molecular interactions by visualizing molecule positions over time while the experimental approach tests interactions profiling the ion transport by concentration. Both approaches are inherently different from determining diffusion coefficients in macroscopic dialysis or permeability cells. The MD simulation studies highly charged $V^{3+}$ ion compared to the studies cited earlier, which modeled water, protons and singly charged ions. The length scale of 30 nm is comparable to the MD simulations and QENS experiments studying water diffusion in ref. [9]. Though protons and water diffusivity has been studied extensively on these tiny length scales also experimentally, the data on V ions come mainly from the already discussed dialysis cells. The work by Intan et al. [11], which looked at vanadium ion sulfonate group interaction cited earlier, modeled a water body with a small molecule of only the side chain ($C_{5}F_{11}SO_{3}^{-}$) of Nafion. In comparison to these, our modeling approach models the entire Nafion membrane at different hydration levels and our experimental approach allows us to quickly detect the influence of small changes in parameter setting, which probably lead to the discrepancies of the diffusion coefficient in the studies cited above.

In summary, we present an experimental approach for measuring vanadium ion diffusion in Nafion membranes using synchrotron micro-XRF concentration profiling. This addresses a key challenge in vanadium redox flow batteries—the vanadium cross-over. It is also a promising methodology for investigating ion transport in ion-exchange membranes in general. Concentration gradients in the micrometer range *through-plane* after short diffusion times are detected and analyzed using a Fickian diffusion model. A direct estimate of the vanadium diffusion coefficient is obtained without having to resort to conventional permeability measurements. By combining molecular dynamics simulations and the sub-micron XRF experiments, macroscopic and molecular insights into ion transport in hydrated PFSA membranes is provided.

## 2. Simulation Method

To construct the configuration of Nafion polymer chains, we use the algorithm proposed by Marchand et al. [28]. A Nafion ionomer is composed of a ${\mathrm{CF}}_{2}$- backbone with pendant sulfonated side chains. The initial geometries of the polymer chains were generated by random walk processes underlying several constraints. The polymer chains have random starting points and each chain has a unique random path. The system comprises 200 Nafion chains periodically separated by ${\mathrm{CF}}_{2}$ groups. The chemical formula for a monomer of the Nafion molecule is:


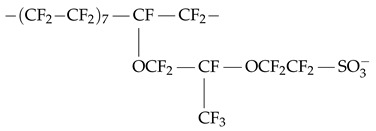


**Figure 1:**
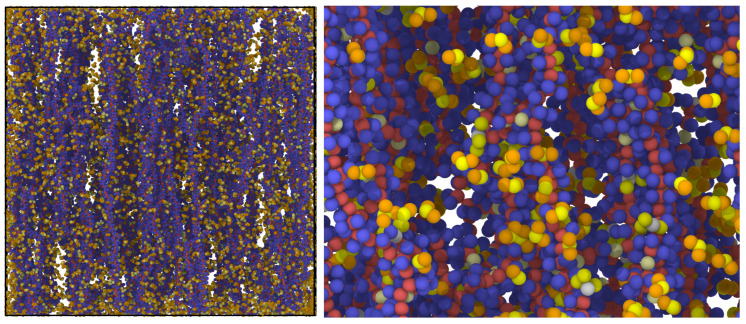
Sketch of a Nafion system (full and zoomed lateral view) with hydration level $\lambda_{H_{2}O/SO_{3}}=6$. Yellow: O, orange: H, red: C, blue: F, light grey: S.

Each polymer chain contains 20 side chains. Thus, the system contains $4000{\mathrm{SO}}_{3}^{-}$ groups. The cubic simulation box has a length of 21 nm. A sketch of the system is shown in Figure 1.

Afterwards, the Nafion system was placed in a cubic box with a number of $H_{2}O$ molecules varying from 8000 to 48,000 corresponding to hydration levels $\lambda_{H_{2}O/SO_{3}}$ from 2 to 12 using the geometry optimization in the Amorphous Cell tool of Materials Studio. In addition, we apply hydronium cations. To understand the effects of vanadium on the local structure, $V^{3+}$ and hydronium, $H_{3}O^{+}$ is added to the system such that the system is neutral ($1333\;V^{3+}$ and one hydronium for $4000\;{\mathrm{SO}}_{3}^{-}$ groups). The setups consist, therefore, of Nafion, water, vanadium $V^{3+}$ and hydronium cations. In a second system, only hydronium are added (4000 hydronium and no $V^{3+}$ such that the system is again neutral). The total number of atoms for the vanadium system for $\lambda_{H_{2}O/SO_{3}}$ = 2 is 281,337, for $\lambda_{H_{2}O/SO_{3}}$ = 6 is 297,337 and for $\lambda_{H_{2}O/SO_{3}}$ = 12 is 321,337. The total number of atoms for the hydronium system for $\lambda_{H_{2}O/SO_{3}}$ = 2 is 296,000, for $\lambda_{H_{2}O/SO_{3}}$ = 6 is 312,000 and for $\lambda_{H_{2}O/SO_{3}}$ = 12 is 336,000. The density of the vanadium system for $\lambda_{H_{2}O/SO_{3}}$ = 2 is 2069 kg $m^{-3}$ and for $\lambda_{H_{2}O/SO_{3}}$ = 6 amounts to 1885 kg $m^{-3}$. This is very close to experimentally measured densities in Nafion 117 of 1990 kg $m^{-3}$ for $\lambda_{H_{2}O/SO_{3}}$ = 2 and 1850 kg $m^{-3}$ for $\lambda_{H_{2}O/SO_{3}}$ = 6 [29].

To describe the Nafion membrane we use the Dreiding force field [30]. For the interaction with vanadium we use the potential parameter set published in [14]. We use the standard Lorentz–Berthelot combining rules for the interactions between different atomic species. The interaction of water and $V^{3+}$ was adopted from Kritayakornupong [31]. The Lennard-Jones parameters for $V^{3+}$ are assigned to the $V^{3+}$ interaction with the TIP3P water model used. The vanadium–vanadium Lennard-Jones parameters have been derived from the London dispersion formula [14]. This force field model has been used to study aqueous solutions and Nafion membranes and has been found to produce consistent findings with experiments [14,32].

The systems are relaxed in a Nosé–Hoover ensemble (NPT) for 1000 ps. The time steps we used were between 0.01 and 1 fs, depending on the system. The reason for this is the difference in van der Waals energy parameters requiring smaller time steps for larger energy values. The simulations are conducted at a temperature of 300 K using a thermostat. The forcite module of Biovia Materials Studio is used in this work to perform the simulations. We apply periodic boundary conditions.

The diffusion coefficient of $V^{3+}$ can be measured from the mean-square displacement of the $V^{3+}$ atoms:

(2) $$D=\frac{1}{2n}\frac{d(\mathrm{MSD})}{dt}$$

where *t* denotes the time, n=3 is the dimensionality of the system and $\frac{d(MSD)}{dt}$ is the slope of the linear region for the mean-square displacement $MSD=\langle |r\left(t\right)-r\left(0\right){|}^{2}\rangle$. Here, r(t) and r(0) represent the atomic positions at time t and initial time, respectively.

## 3. Free Diffusion Experimental Approach and Water Uptake

### 3.1. Chemicals and Instrumentation

Nafion 117 was purchased from Chemours (Wilmington, DE, USA) with a dry membrane thickness of 178 μm and an equivalent weight of 1100 g n(${\mathrm{SO}}_{3}$)^−1^. Sulfuric acid (98%), hydrogen peroxide (30%) and phosphorous pentoxide were obtained from Merck (per analysis, Darmstadt, Germany). Ultra-pure water (>18.2 MΩcm) was prepared by a Purelab Flex 4 system (ELGA Veolia, Paris, France).

Vanadium electrolyte was obtained by electrochemical conversion of $V^{3+}/{\mathrm{VO}}^{2+}$ electrolyte (1.6 M vanadium in 4 M sulfuric acid, Gesellschaft für Elektrometallurgie mbH, Nürnberg, Germany) in an in-house VFB cell described by Lutz and Fittschen [33]. Evaluation of the conversion was obtained using an UV-Vis spectrometer (Analytik Jena, Jena, Germany) with a 1 mm quartz cuvette for the negative electrolyte and a 0.1 mm flow-through quartz cuvette for the positive electrolyte (both Hellma, Müllheim, Germany). An additional evaluation was performed with laboratory X-ray absorption near edge structure (XANES) spectrometry, an easyXES100 (EasyXAFS, Renton, WA, USA, similar to Lutz and Fittschen [33]) and a QuantumLeap Hybrid (Sigray, Concord, CA, USA) were used. The easyXES100 is equipped with a VF-80JM X-ray tube (W/Pd-anode, 25 kV, 3 mA, Varex Imaging, Salt Lake City, UT, USA) and a Ketek VITUS H80 $80\;{\mathrm{mm}}^{2}$ silicon drift detector (Ketek, Munich, Germany). The vanadium speciation was performed with a Ge(422) spherically bent crystal analyzer (XRS Tech, Freehold, NJ, USA). Between two polyimide foils (40 μm thickness, Conrad Electronic, Hirschau, Germany) a 10 mm × 10 mm sheet of quantitative ashless filter paper (blue band, Hahnemühle FineArt GmbH, Dassel, Germany) was placed and soaked with 10 μL electrolyte. The spectra (n = 8) were obtained from 5390 to 5700 eV with the following energy and time steps: 5390–5560 eV, ΔE = 0.25 eV, t = 4 s and 5560–5700 eV, ΔE = 1.0 eV, t = 1 s. Matrix adapt $I_{0}$ was obtained after each specimen measurement. The QuantumLeap Hybrid is equipped with an Eiger2R 500K Si detector (Dectris, Baden, Switzerland) and an X-ray source composed of Mo microstructures embedded in diamond (20 kV, 10 mA). The speciation was performed using a cylindrically curved Si(220) Johansson monochromator crystal (Saint-Gobain, Saint-Pierre-Les-Nemours, France). Evaluation of the XANES was done with Athena [34].

**Figure 2:**
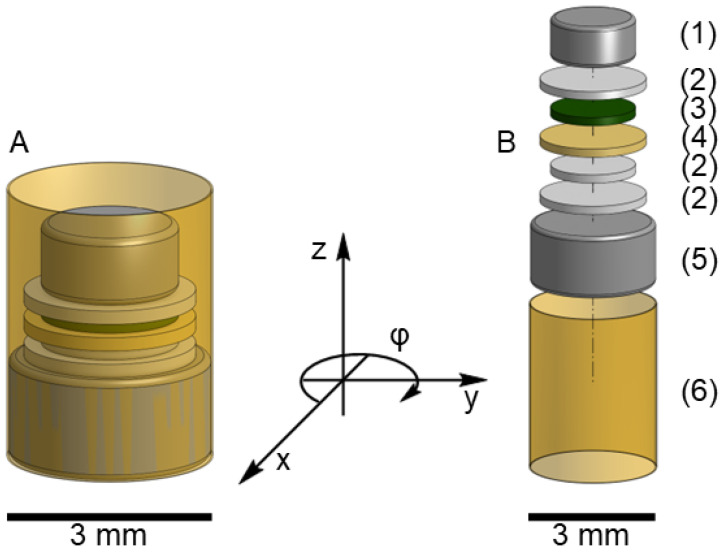
Stacking of the diffusion cell setup with all relevant axes (x, y, z, and φ) following dimensions and structure described by Lutz et al. [37]. (**A**) denotes the full stacked version and (**B**) the exploded view with their own scale, respectively. In the figure, (1) is a neodymium magnet (2 mm diameter), (2) dry filter paper (2 mm and 2.5 mm, respectively), (3) filter paper soaked in green 1.6 M V^3+^ electrolyte (2 mm diameter), (4) hydrated Nafion 117 membrane (2.5 mm diameter), (5) neodymium magnet (3 mm diameter), and (6) Kapton tube (3 mm outer diameter, 2.94 mm inner diameter).

Synchrotron micro-XRF analysis was performed at the Hard X-ray Micro/Nano-Probe beamline P06 (PETRA III, DESY, Hamburg, Germany) [35]. Measurements were performed in fluorescence geometry with a single element $50\;{\mathrm{mm}}^{2}$ SII Vortex EM Si-drift detector and a SII Vortex ME4 four-element SDD (both Hitachi High-Tech, Chatsworth, CA, USA). Both detectors were equipped with aluminum collimators and were set at angles of −45° and 45°, respectively, relative to the incoming beam at approx. 20 mm distance to the sample. Monochromatization was achieved with an Si(111) double-crystal monochromator with an energy resolution of ΔE/E = 2 × $10^{-4}$. Horizontally, deflecting Si mirrors were used for higher harmonic suppression. The beam was focused with one mirror of a Kirkpatrick–Baez mirror system (JTEC, Saito-Yamabuki, Osaka, Japan) to 0.5 μm vertically while being unfocused horizontally. The horizontal size was limited to 100 μm with slits. Sample movement was done with a hexapod (Newport, Irvine, CA, USA) with a magnetic mount on top allowing fast placement of the sample. The sample was cooled to 120 K with a Oxford Cryostream Cooler 700 (Oxford, United Kingdom) from above to stop the diffusion during the measurements. A digital microscope with an HV-Z50W lens (Keyence, Osaka, Japan) was set between the sample and the Kirkpatrick–Baez mirror system. Micro-XRF measurements were performed as two-dimensional mapping of rotation φ and vertical translation *z* in order to spread incident radiation and reduce photo-oxidation. The scan was carried out with continuous movement of the fast axis (*z*) in combination with rotation (φ) as the slow axis (see Figure 2). The mapping was done at 5700 eV with a dwelling time of 10 ms per pixel (0.5 μm × 100 μm spot size, steps of 0.5 μm in z and rotation of φ=6°, respectively).

The resulting data array with 9 times 1000 data points (rotation φ and z-axis) was evaluated with Python 3.12.7 using the Spyder IDE package (version 5.5.1) for a region of interest of the XRF spectra for V $K_{\alpha }$. The line scans were aligned to the static diffusion front and then summed up to a single line scan for further evaluation.

### 3.2. Sample Preparation

Nafion 117 was pre-treated similar to Tang et al. [36]. The membranes (1 × $5\;{\mathrm{cm}}^{2}$) were subsequently handled for 1 h at 80 °C in 3 w% hydrogen peroxide, ultra-pure water, 1 M sulfuric acid and ultra-pure water. The pre-treated membranes were stored in ultra-pure water before usage.

The diffusion cells were constructed according to Lutz et al. [37]. A filter paper (2.5 mm diameter) was soaked with $V^{3+}$ electrolyte and held in liquid nitrogen. Inside a Kapton tube (3 mm outer diameter, 2.94 mm inner diameter) a stacked system (bottom to top) consisting of a Ni-coated Nd magnet (3 mm diameter), a dry filter paper (2.5 mm diameter), a dry filter paper (2 mm diameter), hydrated Nafion 117 (2.5 mm diameter, thickness of about 180 μm), filter paper with $V^{3+}$ (2 mm diameter), a dry filter paper (2.5 mm diameter) and a Ni-coated Nd magnet (3 mm diameter) was constructed (see Figure 2). The stacking was performed in liquid nitrogen. After construction the diffusion cell was allowed to heat up to room temperature for 600 s before cooling in liquid nitrogen, again, and transferred in liquid nitrogen to the experiment. The cryostream cooler is placed approx. 10 mm above the measurement position ensuring 120 K at the sample and preventing buildup of ice due to the dry nitrogen flow.

### 3.3. Water-Uptake

For water-uptake experiments, pretreated membranes of 1 × $5\;{\mathrm{cm}}^{2}$ were dried subsequently for 30 min in air, for 12 h in a desiccator over phosphorous pentoxide, and at 80 °C and 100 mbar in an vacuum drying oven. The dried membranes were soaked in 10 mL of ultra-pure water, 0.9 M, 1.8 M, 2.7 M, 3.6 M, and 4.5 M sulfuric acid, respectively, for 72 h. The hydrated membranes were taken out of the solution and the superficial solution was removed with laboratory wipes. The mass of the dried membrane $m_{dry}$ and of the hydrated membrane $m_{hydr}$ were determined to obtain the ratio of water to the sulfonic acid groups $\lambda_{H_{2}O/SO_{3}}$. With $M_{H_{2}O}=18$ g ${\mathrm{mol}}^{-1}$ the $\lambda_{H_{2}O/SO_{3}}$ is calculated as follows:

(3) $$\lambda_{H_{2}O/SO_{3}}=\frac{\left(m_{hydr}-m_{dry}\right)1100gn{\left({\mathrm{SO}}_{3}\right)}^{-1}}{m_{dry}M_{H_{2}O}}$$

For each sulfuric acid concentration and ultra-pure water six replicates were studied.

### 3.4. Free Diffusion Experiment

The diffusion coefficient of $V^{3+}$ in fully hydrated Nafion 117 is determined by interpreting the concentration profile obtained by a free diffusion experiment through the approx. 180 μm thick membrane. On one side the membrane is exposed to a 1.6 M $V^{3+}$ solution in 4 M sulfuric acid, which is a typical VFB electrolyte. The electrolyte is soaked in filter paper and functions as an infinite reservoir for the vanadium ions. The membrane is prepared as described above with a final soaking in ultra-pure water. The back side is also covered by filter paper. The setup is assembled inside a Kapton tube and fixated with two magnets, details of the stack can be found in [37]. The stack is assembled in liquid nitrogen and allowed to defrost for 600 s. Vanadium concentration profiles of the refrozen cells were then recorded by synchrotron micro-XRF at the Hard X-ray Micro/Nano-Probe beamline P06 at DESY. The beamline and data collection and processing details are found above. From the profiles at 600 s diffusion duration average diffusion coefficients were obtained. The procedure is similar to Malhotra et al. [38], where the authors used a scanning electron microscope and energy dispersive X-ray detection (SEM-EDX) to obtain the profiles of Hg concentration for studying the diffusion of Hg in Ag Sn-compounds. The authors use the formalisms of unsteady diffusion in a semi-infinite slab. Although theirs as well as our system are not infinite with respect to the spatial dimension, they become quasi semi-infinite for slow diffusion and/or short observation times. The error function erf(z) approaches unity between approx. z=1.8 to *∞*. Here erf(z) equals $1-C/C_{s}$ and we need to decide which deviation from unity is acceptable. Liebhafsky [39] and later Jain [40] studied the boundaries of the semi-infinite slab system in detail. The latter applied a 1% condition for the fractional concentration change as threshold for treating a finite slab as an semi-infinite slab. A threshold of 0.08 = $D\cdot t/x^{2}$ was derived. We applied also the 1% condition and accept a concentration reduction, so $C/C_{s}$ becomes 0.01 and the erf(z) becomes 0.99 as criterion that the slab can be considered semi-infinitely thick. Using this criterion the minimal thickness or the maximum diffusion time or the maximum diffusion coefficient can be calculated from 0.075 = $D\cdot t/x^{2}$ ($D=x^{2}/4t{\left(\left[erf^{-1}\left(1-C\right)/C_{s}\right]\right)}^{2},1-C/C_{s}=0.99,erf^{-1}\left(1-C\right)/C_{s}=1.82139)$. Using this threshold and the experimentally determined D of $4\times 10^{-13}\;m^{2}s^{-1}$ and 600 s diffusion time we obtained 57 μm as minimal thickness (6075 s as maximum diffusion time and 4.05 × $10^{-12}\;m^{2}s^{-1}$ the maximum diffusion coefficient). The 180 μm thick membrane exceeds this thickness, hence, it can be regarded as semi-infinite slab. The one-dimensional concentration profile for unsteady diffusion in a semi-infinite slab is given by:

(4) $$C\left(x,t\right)=C_{s}erfc\left(\frac{x}{\sqrt{4Dt}}\right)+C_{0}erf\left(\frac{x}{\sqrt{4Dt}}\right)$$

Here, *x* is the distance into the membrane. The surface is located at x=0. $C_{S}$ is the $V^{3+}$ concentration at the surface but inside the membrane which is considered constant. It is given by the partitioning coefficient $K_{V^{3+}}$ times the concentration in the electrolyte $C_{E}$ ($C_{E}$: 1.6 M). This coefficient is not needed for the determination of the diffusion coefficient in this experiment. The interpreted profile starts inside the membrane, where the concentration is already lowered due to partitioning. The position of $C_{s}$ can be determined quite precisely, with in a range of 0.5 μm. The procedure is described in detail in Section 4.2. and in the Supplementary Materials. For the benefit of comparison with the literature, the assumption can be made that $C_{s}$ equals the concentration which is achieved inside the membrane, when it is equilibrated in the electrolyte for long time. According to our previous results, the uptake of $V^{3+}$ is $\lambda_{V^{3+}/SO_{3}}$= 0.137 which corresponds to a vanadium concentration of 0.104 mol ${\mathrm{kg}}^{-1}$ of the hydrated membrane (molality; $\lambda_{H_{2}O/SO_{3}}$ = 12) [41]. The partitioning coefficient, therefore, is $K_{V^{3+}}$ = 0.09 corresponding to the concentration of vanadium in the entire hydrated membrane (details on the computation and the concentrations in the membranes water body are given in the Supplementary Materials). The boundary conditions are $C\left(x=0\right)=C_{s}$ = $K_{V^{3+}}$ 1.18 mol ${\mathrm{kg}}^{-1}$= 0.104 M and $C_{0}$ is the initial concentration inside the membrane at $t=0\;C\left(x=\infty \right)=C_{0}$ = 0.

(5) $$C\left(x,t\right)=C_{s}\cdot erfc\left(\frac{x}{\sqrt{4Dt}}\right)=C_{s}\cdot \left[1-erf\left(\frac{x}{\sqrt{4Dt}}\right)\right]$$

If we solve for the diffusion coefficient we need to apply the anti-error function yielding $x/\sqrt{4Dt}$. Assuming a constant diffusion coefficient D the following linear model is obtained which describes the dependence between the inverse error function and x being the variables:

(6) $$erf^{-1}\left(\left(C_{s}-C\right)/C_{s}\right)=\frac{1}{\sqrt{4Dt}}\cdot x$$

Plotting ${\mathrm{erf}}^{-1}\left(1-C/C_{s}\right)$ on the y-axis and x on the x-axis ${\sqrt{4Dt}}^{-1}$ becomes the slope and the diffusion coefficient can be calculated as follows:

(7) $$D={\left(\frac{d(erf^{-1}\left(1-C/C_{s}\right))}{dx}\right)}^{-2}\cdot \frac{1}{4t}$$

The diffusion depth at 1/2$\cdot (C_{s}-C_{0})$ may also be used for the determination of the diffusion coefficient. The depth at half of the maximum concentration equals $\sqrt{Dt}$. Here, however, interpreting the entire profiles with the error function was favoured because of the otherwise two-point calibration. The concentrations $C_{s}$ and C(x,t) are considered proportional to the integrated counts from the V $K_{\alpha }$ line and were used instead of concentrations. This is eligible when matrix effects —mainly absorption—are constant, the sample thickness is infinite and the matrix absorption does not change over the probed concentration range. Then, the variation of net counts only depends on a change in concentration ($C_{i}$) because all other parameters remain constant over the measurement. The net counts ($N_{i}$) of an elemental line are then described as follows [42] with α including the constant matrix effects:

(8) $$N_{i}=\alpha \cdot C_{i}\cdot N_{0}\cdot \tau_{\left(E,Z\right)}\cdot \left(\left(s-1\right)/s\right)\cdot \omega_{fluo}\cdot P_{\left(K_{\alpha }/\sum K\right)}$$

The parameters are the geometries of the setup and the sensitivity of the detector as well as constant matrix effects α, the X-ray source counts $N_{0}$, the photoelectric cross section τ, the proportion absorbed by the shell of interest (here K-shell) given by the edge-jump (s−1)/s, the fluorescence yield $\omega_{fluo}$, and the probability P that a $K_{\alpha }$ photon is emitted. The X-ray exciting beam scans the membrane through plane, meaning a profile over the 180 μm thick membrane is obtained. In the other direction the membrane is extending approx. 3 mm. We assume that the vanadium concentration in that direction is constant and the 3 mm well exceed the information depth of the sample for the vanadium Kα radiation. The matrix effects can also be considered constant with changing vanadium concentration. The information depth of the material at a concentration of $\lambda_{V^{3+}/SO_{3}}$= 0.137 and three orders of magnitude lower, i.e., 0.000137, were calculated to be 76.9 μm and 77.4 μm which results in a difference of less than 1%. The calculation are documented in the Supplementary Materials using fundamental parameter from McMaster et al. [43]. It can be concluded that the absorption is not changing significantly, due to the change of concentration in the 180 μm profile and that the 3 mm in the other direction can be considered of infinite thickness. The matrix effects are approx. constant and the fluorescence signal scales as described in Equation (8) with the vanadium concentration.

## 4. Results

### 4.1. MD Simulation

**Figure 3:**
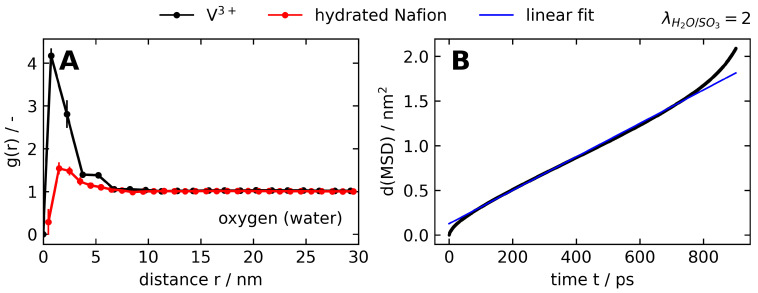
(**A**) visualizes the simulated radial distribution function g(r) between oxygen atoms of two water molecules for $\lambda_{H_{2}O/SO_{3}}=2$. The error bars denote the absolute error from block averaging of five different simulation blocks at 500–900 ps. (**B**) displays the mean-square displacement (MSD) of the $V^{3+}$ atoms (black line) along with the linear fit (blue line).

We examine the molecular structure after relaxation by means of a coordination analysis implemented in OVITO 3.15.4 [44]. The radial distribution function g(r) describes the (short-range) order in a material. Assume atom 1 is located at position ${\vec{r}}_{1}$. The average number of atoms in a small volume $d^{3}r_{2}$ around position ${\vec{r}}_{2}$ is proportional to the size of the volume $d^{3}r_{2}$ and the average atom number density *n*. Therefore, the number of atoms can be written as follows: $ng\left(r\right)d^{3}r_{2}$. The dimensionless proportionality factor, g(r), in this relation, is the radial distribution function. In Figure 3A, the radial distribution functions g(r) between the oxygen atoms of two water molecules are plotted for the samples with and without $V^{3+}$ for $\lambda_{H_{2}O/SO_{3}}$ = 2, where we find the largest differences. The error bars denote in the figure are the absolute error from block averaging of five different simulation blocks at 500–900 ps. The relative standard error of the linear fit is 0.2%. The limited number of fully processed MD data points does not allow to construct a robust sensitivity analysis with respect to water content and temperature. The presence of vanadium increases the probability of finding water molecules at a short distance which generates an ordering effect. The same can be seen for $\lambda_{H_{2}O/SO_{3}}$ = 6 (Supplementary Figure S2). Our simulations for the highest hydration level shows that the ordering effect of vanadium ions on water molecules becomes less visible. These results are shown in Supplementary Figure S3. The presence of $V^{3+}$ also seems to have an ordering effect on the ${\mathrm{SO}}^{3-}$ groups and the water molecules for $\lambda_{H_{2}O/SO_{3}}$ = 2 and 6 (Supplementary Figures S1 and S2). Interestingly, at $\lambda_{H_{2}O/SO_{3}}$ = 12 the ordering (which is visible) is independent of the presence of $V^{3+}$ (Supplementary Figure S3).

The differences in the radial distribution functions g(r) of the sulfur atoms are very small. The ${\mathrm{SO}}^{3-}$ groups may move on average closer to each other when vanadium is present, which was suggested by previous MD studies [14]. However, in this simulation, the differences are not significant. Interestingly, at $\lambda_{H_{2}O/SO_{3}}$ = 12 the positions of the ${\mathrm{SO}}^{3-}$ groups relative to each other is more constant than for the lower hydration states (Supplementary Figure S3).

**Figure 4:**
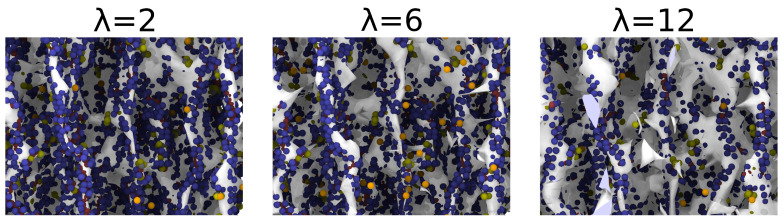
Snapshots of hydrated Nafion membranes at $\lambda_{H_{2}O/SO_{3}}=2$, $\lambda_{H_{2}O/SO_{3}}=6$ and $\lambda_{H_{2}O/SO_{3}}=12$. Yellow: O, orange: H, red: C, blue: F, light grey: S. The water molecules are shown as white isosurface.

To visualize the water network morphology, simulation box snapshots are shown in Figure 4. Isolated water clusters can be seen at lower hydration level $\lambda_{H_{2}O/SO_{3}}$ = 2 and $\lambda_{H_{2}O/SO_{3}}$ = 6. At high hydration these clusters are larger and we observe a more interconnected hydrophilic network.

To understand the transport properties we calculate the diffusion coefficient of $V^{3+}$. We consider here the diffusion coefficient for $\lambda_{H_{2}O/SO_{3}}$ = 6, which is an intermediate water content.

Note that the diffusion data for ionic diffusion exhibit statistical errors that can be quantified by studying many statistically equivalent simulations. However, due to the high computational effort we performed only one simulation per system setup.

Figure 3B shows the MSD of the $V^{3+}$ atoms for $\lambda_{H_{2}O/SO_{3}}$ = 2. The linear fit yields a diffusion coefficient of $3.1\times 10^{-10}\;m^{2}s^{-1}$ with a correlation coefficient $R^{2}=0.986$. Note that we see a nonlinear behavior at higher simulation times. For $\lambda_{H_{2}O/SO_{3}}$ = 6 we find $2.5\times 10^{-10}\;m^{2}s^{-1}$ with a correlation coefficient $R^{2}=0.994$ and for $\lambda_{H_{2}O/SO_{3}}$ = 12 we find $2.7\times 10^{-10}\;m^{2}s^{-1}$ with a correlation coefficient $R^{2}=0.997$. The increase in D, due to increasing water content appears low; however, even the Ds of water only increase slightly, e.g., in ref. [9] local water diffusion coefficients determined by QENS were $0.7\times 10^{-9}\;m^{2}s^{-1}$ at $\lambda_{H_{2}O/SO_{3}}$ = 4.7 and $1.0\times 10^{-9}\;m^{2}s^{-1}$ at $\lambda_{H_{2}O/SO_{3}}$ = 10. In comparison with water diffusion, the multivalent $V^{3+}$ ion is expected to be strongly bound to the charge group of the polymer. These ions are, thus, dehydrated and the diffusivity is rather invariant with changes in the water content of the membrane. Note that diffusion is also concurrent with time-dependent processes like swelling and polymer relaxation.

### 4.2. Free Diffusion Experiment

**Figure 5:**
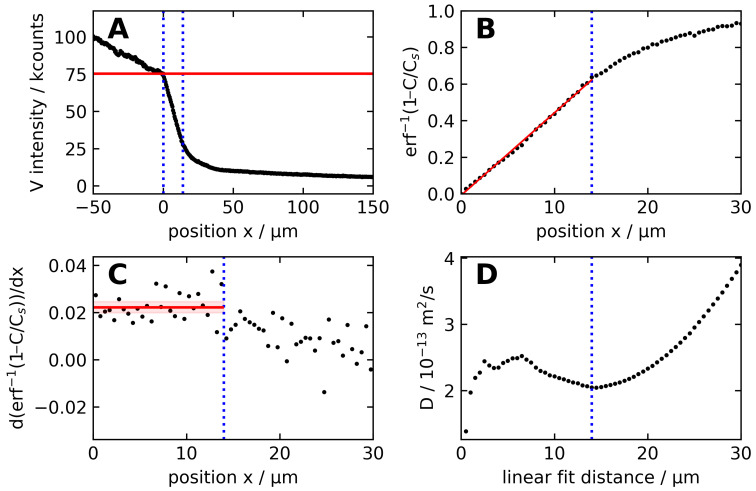
(**A**) denotes the $V^{3+}$ profile from micro-XRF measurements (black line) for a diffusion time of 600 s. The red line indicates the starting point of the diffusion while the area between both blue dashed lines corresponds to the linear range of the inverse error function. Individual measurements lines were aligned and merged to create the profile. (**B**) shows the inverse error function versus position according to Equation (7) from the merged data (black dots). The red line denotes the fit while the area between the blue dashed line and the y-axis corresponds to the linear range of the inverse error function. From the slope, the diffusion coefficient D is determined. (**C**) denotes the first derivative of the inverse error function $erf^{-1}\left(1-C/C_{s}\right)$ after the position *x* (black dots) with mean value (red line) and 95% confidence interval (light red band). The linear fit range shown in (**B**) is given with the blue dashed line. (**D**) visualizes the influence of the fitting range onto the diffusion coefficient D (black dots). The blue dashed line indicates the linear fit range shown in (**B**).

The free diffusion experiment yields the $V^{3+}$ profile shown in Figure 5A. The profile is obtained by merging the available line scans to provide sufficient counts for an acceptable error with respect to counting statistics (a relative error of ca. 0.6% is obtained at the lowest count rate used in the regression). The profile corresponds well to a semi-infinite diffusion approach. The zero position and the intensity corresponding to $C_{s}$ is determined by fitting a line with a constant slope towards the reservoir and one with a steep slope inside the membrane to the data. The point where both lines intersect is set as $C_{s}$ position.

The intersection can be calculated with the following equation using the slopes ($m_{i}$) and intersections ($b_{i}$) of the two linear fits:

(9) $$x=\frac{b_{2}-b_{1}}{m_{1}-m_{2}}$$

The absolute error is estimated from error propagation of the standard errors for each parameter ($\sigma_{i}$) and is given as:

(10) $$\sigma_{x}=\sqrt{\frac{\sigma_{b_{1}}^{2}+\sigma_{b_{2}}^{2}}{{\left(b_{2}-b_{1}\right)}^{2}}+\frac{\sigma_{m_{1}}^{2}+\sigma_{m_{2}}^{2}}{{\left(m_{1}-m_{2}\right)}^{2}}}\cdot x$$

The derivation of the intersection and the errors are described in detail in the Supplementary Materials. This method is commonly used in conductometry titration yielding reproducible results with small error margins [45,46].

**Figure 6:**
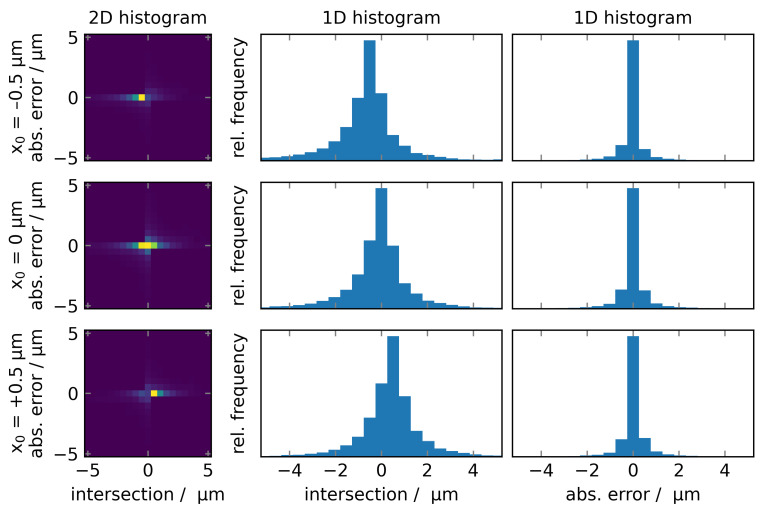
Histograms from interception determination for correct $C_{s}$ determination as well as an offset of ±0.5μm. The 2D histograms for the interception and the absolute error are shown as well as the 1D histograms for each of the mentioned. Each histogram is shown with bins of 0.5 μm.

Applying that principle provides $C_{s}$ position with accuracy better than the scans step size of 0.5 μm. The linear ranges can be approximated by the 1st derivative and are most critical for the diffusion profile due to fewer data points than the reservoir. As the fitting range may seem arbitrarily set we evaluated all possible combinations (see Supplementary Figure S4) to show that the exact fitting range has only a minor influence on the determined intersection. The fit ranges are set to −50 μm to 2 μm for the first fit and −2 μm to 16 μm for the second. With those borders the linear range is definitely included in the respective fits. The resulting histograms of the intersection show a normal distribution around $x_{o}=0$ corresponding to our $C_{s}$ position (see Figure 6). If there would be an offset of one data point in the $C_{s}$ position, this is clearly visible in a shift of the center of distribution. The propagated error distribution remains unchanged. The sensitivity of this method allows us to determine the position of $C_{s}$ with an uncertainty of a single data point (0.5 μm). To match the experimental data, the bin size was chosen to be 0.5 μm.

As shown in Equation (7) free diffusion can be described using an inverse error function formalism. The data is plotted accordingly, i.e., the inverse error function versus the distance into the membrane (Figure 5B). In the diffusion zone extending from 0 to 14 μm the plot follows very precisely an error function. From its linear range the slope is determined (see Equation (7)). The linearity in this range was evaluated by plotting the first derivative of the data shown in Figure 5B. The slope is not significantly different from zero in the indicated range (dotted blue line Figure 5C). The 95% confidence interval of the mean of the first derivative obtained from it is 4.5 × $10^{-2}\;\pm$ 2.3 × $10^{-3}$ (red line: mean of the 1st derivative in Figure 5C with 95% confidence interval).

From the experimental data an average diffusion coefficient of $4\times 10^{-13}\;m^{2}s^{-1}$ at a $\lambda_{H_{2}O/SO_{3}}=12$ and ca. 20 °C was calculated, which is a little smaller than the smallest literature data ($7\times 10^{-13}\;m^{2}s^{-1}$); however, this is significantly smaller than the simulated diffusion coefficient ($2.5\times 10^{-10}\;m^{2}s^{-1}$ at $\lambda_{H_{2}O/SO_{3}}$ = 6). The relative error of the diffusion coefficient was 1.8% determined using propagation of errors. The diffusion coefficient determined here is derived from the optimal parameters of the evaluation and influenced by (a) proper linear fit range and (b) correct $C_{s}$ position. Figure 5D visualizes the influence of the proper linear fit range on the optimal $C_{s}$ position. With too few data points statistical errors have a strong influence while for too large ranges the non-linearity distorts the fit. Allowing relative errors up to 10% for the fitting range will result in up to 2.9% difference. Errors in determining $C_{s}$ are assumed with up to ±0.5 μm due to the good accuracy of the intersect method. The reported errors are the maximum values resulting from both error sources, changes in linear fit range and $C_{s}$ position. Calculating the diffusion coefficient gives up to 4.4% error. Even by strong miscalculation of $C_{s}$ by 1.5 μm the maximum error is 11.7%. A concluding error calculation for difference $C_{s}$ positions with varying fitting ranges is given in Supplementary Figure S5 as well as Supplementary Table S4. The method is, therefore, suitable for determining acceptable diffusion coefficients from single micro-XRF scans with short measurement time.

## 5. Discussion

The diffusion of $V^{3+}$ ions inside Nafion 117 was studied on a molecular level and on the micro meter scale. MD simulations were applied as well as the evaluation of the vanadium profile obtained using micro-XRF in a free diffusion experiment. Diffusivity of ions inside ion-exchange membranes is significantly influenced by the established intermolecular interactions. Our MD simulation results suggest an ordering interaction of $V^{3+}$ inside the membrane at low levels of hydration (see Figure 3A). The ions probably keep the water molecule on average closer together. At higher hydration levels, this effect is no longer significant. It was suggested that higher valent ions have ionic interaction with more than one sulfonic acid group [1], this is not significantly shown in the simulations. The average amount of sulfur atoms in close distance may be higher if $V^{3+}$ is present but the radial distribution function is too noisy for that call.

The local displacement observed in the MD simulation yielded a diffusion coefficients of $2.7\times 10^{-10}\;m^{2}s^{-1}$, $\lambda_{H_{2}O/SO_{3}}$ = 12, 27 °C. This is ca. three orders of magnitude higher than the micro meter scale diffusion coefficient obtained from the experimentally derived profiles of $4\times 10^{-13}\;m^{2}s^{-1}$ at a $\lambda_{H_{2}O/SO_{3}}=12$ and ca. 20 °C. This decrease is in line with previous observations for water in Nafion, where effective diffusivities on the micrometer scale (about 1 μm) are typically one to two orders of magnitude lower than local nanometer-scale values [9]. In this work diffusivity of a multivalent ion over an even larger length scale was observed. Zhang et al. [10] described that the mobility of the molecules on the nanoscale are not the rate limiting step. They studied monovalent ions in hydrated (37% $H_{2}$O) PDAC/PSS membranes by MD simulation and found that trapping is poorly described by considering only steric barriers. They suggest a large influence of electrostatic trapping, which can be expected to have an even larger influence on multivalent ions and should also be relevant in the pure cation exchange polymer electrolyte membrane Nafion.

The displacement in the MD simulation is obtained on a short length scale only affected by electron potential interactions and Brownian motion. However, there may be other reasons that the displacements are faster in our MD simulations. Comparing the water diffusion coefficient, from our MD models with the one run by Berrod et al. [9], ours is ca. seven times higher with $D_{H_{2}O}$ = $7\times 10^{-9}\;m^{2}s^{-1}$ at $\lambda_{H_{2}O/SO_{3}}$ = 2 than Berrods et al. which is $0.4\times 10^{-9}\;m^{2}s^{-1}$ at $\lambda_{H_{2}O/SO_{3}}$ = 2.8. They also found a decrease of diffusion coefficient of about one order of magnitude when changing from the nanometer range to the micro meter scale. This effect was even more pronounced with other types of membrane with declines of D by two orders of magnitude.

The experimentally derived vanadium ion diffusion coefficient of $4\times 10^{-13}\;m^{2}s^{-1}$ at a $\lambda_{H_{2}O/SO_{3}}=12$ and ca. 20 °C seems to be somewhat low even if a sub-diffusion is expected. Comparing it to the dialysis cell experiments, the D of $V^{3+}$ closest to our value is the one determined by Elgammal et al. [7] in Nafion 117 e.g., D = $7\times 10^{-13}\;m^{2}s^{-1}$, which was obtained at 30 °C and a water content of $\lambda_{H_{2}O/SO_{3}}=11$. Depending on how the dialysis experiments are designed, osmosis and additional potentials, e.g., Donnan potentials can increase the concentration changes and will yield a macroscopic diffusion coefficient. The determination of the membrane area and void fractions may also give rise to errors. Additionally, Ds obtained from the dialysis may either be derived by considering an effective membrane area, or the partitioning of the ions between the reservoir and the membrane. This is not always described in detail. Note that thermodynamic and interfacial effects influencing mobilities in macroscopic cells do not appear in the MD self-diffusion coefficient. If this is considered as in, e.g., Darling et al. [47], diffusion coefficient of $V^{3+}$ ions inside Nafion 115 becomes $0.62\times 10^{-10}\;m^{2}s^{-1}$. The highest value they report is for the ${\mathrm{VO}}_{2}$^+^ species with $3.30\times 10^{-10}\;m^{2}s^{-1}$. It should be noted that another study found a higher D for $V^{3+}$ compared with ${\mathrm{VO}}_{2}$^+^. Additionally, our experiment studied unsteady diffusion while dialysis cells are quasi in a steady state. So the differences may results from these experimental differences. In conclusion, the profile obtained here are well described with a Fickian model derived error function. This approach—because of the short diffusion time—probes mainly the vanadium ion diffusion into a vanadium free membrane. Interestingly, it is significantly slower than the diffusion in the vanadium equilibrated membrane, which is probed in the dialysis approach.

To summarize, the MD-derived diffusivity and the micro-XRF-derived diffusivity do not measure exactly the same transport process. The MD simulations probe local self-diffusion on nanometer length scales and nanosecond times, in a homogeneous segment of a hydrated membrane. In contrast, the micro-XRF experiment probes effective transport over micrometer distances and hours (with respect to the critical diffusion time of 1.7 h for the definition of the semi-infinite slab), in a real membrane with morphological and chemical heterogeneities.

## 6. Conclusions

In summary, the MD simulation approach allows studying the interaction of the highly charged vanadium ions with water molecules and ion exchange groups inside ionomeric membranes on short length scale. We can conclude on an ordering effect of the vanadium ions at low levels of membrane hydration. The diffusion coefficient determined from the MD simulation considering no other driving forces than electron interaction potentials and temperature motion are considerably higher than those from steady-state diffusion in permeability cells [5,6,7] and higher than the diffusion coefficient obtained from unsteady diffusion into vanadium ion free environment. The experimental approach developed here studies ion mobility on a shorter length than the common cell approaches, but considerably longer than the MD simulation. Whilst unsteady diffusion into a vanadium free environment is studied, the diffusion is well described by Ficks second law. The procedure established here is promising to study diffusion microscopically inside ionomeric membranes. Differences of the ion selectivity with the specific ion-exchange polymer is sensitively probed in our short time unsteady state experiment. Temperature-controlled experiments are realizable in the future as well as diffusion into membranes equilibrated with counter ions other than $H^{+}$. A benefit of the approach is its independence from partitioning and void fractions of the individual membrane and the accessibility of unsteady state processes. For now, the membrane was probed in a *through-plane* direction with small micro-XRF spot sizes (0.5 μm). Adaption of the procedure to lab-based micro-XRF in an *in-plane* direction can facilitate studies on several parameters influencing membrane diffusion in a very quick manner.

## Data Availability

An initial draft of the manuscript is published in the PhD thesis of S.H. [48]. Data not given in the main paper and the Supplementary Materials is available at request, please contact the corresponding author.

## Supplementary Materials

The following supporting information can be downloaded at: https://www.mdpi.com/article/10.3390/membranes16060195/s1, Table S1: Vanadium concentration partitioning coefficients at $\lambda_{H_{2}O/SO_{3}}=12$, a electrolyte concentration of 1.6 M (ρ = 1.36 g ${\mathrm{cm}}^{-3}$) and a density of the hydrated membrane of $\rho_{Nafion}$ = 1.98 g ${\mathrm{cm}}^{-3}$; in the water phase of the membrane (top) and in the membrane (bottom); Table S2: Attenuation coefficients and weighted fractions for $\lambda_{H_{2}O/SO_{3}}=12$, a density of the hydrated membrane of $\rho_{Nafion}$ = 1.98 g ${\mathrm{cm}}^{-3}$, and two concentration of vanadium either $\lambda_{V^{3+}/SO_{3}}=0.137$ or $\lambda_{V^{3+}/SO_{3}}=0.000137$ (bottom) and the dry membrane (top); Table S3: Linear attenuation coefficients $\mu_{lin}$ and information depths of $\lambda_{H_{2}O/SO_{3}}=12$ for both vanadium concentrations of $\lambda_{V^{3+}/SO_{3}}=0.137$ and a $\lambda_{V^{3+}/SO_{3}}=0.000137$ in the membrane; Figure S1: Simulated radial distribution function g(r) for $\lambda_{H_{2}O/SO_{3}}=12$. (**A**) between oxygen atoms of two water molecules, (**B**) between the sulfur atoms of different Nafion molecules, (**C**) between sulfur and oxygen atoms, and (**D**) between water and vanadium atoms; Figure S2: Simulated radial distribution function g(r) for $\lambda_{H_{2}O/SO_{3}}=6$. (**A**) between oxygen atoms of two water molecules, (**B**) between the sulfur atoms of different Nafion molecules, (**C**) between sulfur and oxygen atoms, and (**D**) between water and vanadium atoms; Figure S3: Simulated radial distribution function g(r) for $\lambda_{H_{2}O/SO_{3}}=12$. (**A**) between oxygen atoms of two water molecules, (**B**) between the sulfur atoms of different Nafion molecules, (**C**) between sulfur and oxygen atoms, and (**D**) between water and vanadium atoms; Figure S4: Fit results for two linear functions in the range from −50 μm to 2 μm and from −2 μm to 16 μm, respectively; Figure S5: Influences of errors in determining the position for $C_{s}$ on (**A**) the inverse error function and (**B**) subsequently on the diffusion coefficient D. The linear range for the optimal case is denoted with the blue dots in both subplots; Table S4: Determined diffusion coefficients *D* allowing errors in position determination of $C_{s}$ and fitting range.
