# Supplementary material for: Understanding Vanadium Ion Diffusion in Nafion Using an Atomistic Study and Microscopic Concentration Profiles
Source: Membranes (Basel). 2026 Jun 3;16(6):195. doi: 10.3390/membranes16060195 (PMC13302885; doi:10.3390/membranes16060195)
Supplement: Supplementary file 1 [file membranes-16-00195-s001.zip › membranes-4201572-supplementary.pdf]

Supplementary Materials: Understanding vanadium ion diffusion in Nafion using an atomistic study and microscopic concentration profiles

Sven Hampel [1,2], Christian Lutz [1], Gerald Falkenberg [2], Joanna Kolny-Olesiak [1], Ursula E.A. Fittschen [1] and Nina Merkert [3,]*

1. Vanadium concentration and partitioning coefficient

The concentration of V^{3+} in the hydrated membrane was determined in [1] using TXRF and ICP-OES to be $\lambda_{V^{3+}/SO_3} = 0.137$. The concentration of water was determined as described in the main document (C. Water-uptake) to be $\lambda_{H_2O/SO_3} = 12$. This value was used for all calculations. The water volume per $n(SO_3)$ was calculated considering the molecular weight, n$(H_2O) = 12$ and a density of water of 1 g cm^{-3}. The equivalent weight (EW) used is 1100 g n(SO_3)$^{-1}$ and density of the hydrated membrane is 1.98 g cm^{-3} according to the manufacturer (Nafion 117, Chemours, Wilmington, Delaware, USA). The concentration of the electrolyte (C_e) was 1.6 M with a density of ρ = 1.36 g cm^{-3}. From this values concentrations and partitioning coefficients were calculated:

$$K_{membrane} = C_m / C_e$$

$$EW(12\ \lambda_{H_2O/SO_3}) = EW(1100\ g\ n(SO_3)^{-1}) + 12\ mol_{H_2O}/mol_{SO_3} \cdot M_{H_2O}$$

$$C_m(12\ \lambda_{H_2O/SO_3}) = 0.137\ \lambda_{V^{3+}/SO_3} \cdot 1000\ g\ L^{-1} / EW(12\ \lambda_{H_2O/SO_3})$$

$$C_e = 1.6\ mol\ L^{-1} / \cdot 1.36\ kg\ L^{-1} = 1.18\ mol\ kg^{-1}$$

$$K_{waterbody} = C_w / C_e$$

$$C_w(12\ \lambda_{H_2O/SO_3}) = 0.137\ \lambda_{V^{3+}/SO_3} \cdot 1000\ g\ L^{-1} / (12\ mol_{H_2O}/mol_{SO_3} \cdot M_{H_2O})$$

$$C_e = 1.6\ mol\ L^{-1}$$

Table S1. Vanadium concentration partitioning coefficients at $\lambda_{H_2O/SO_3} = 12$, a electrolyte concentration of 1.6 M (ρ=1.36 g cm^{-3}) and a density of the hydrated membrane of ρ_{Nafion}=1.98 g cm^{-3}; in the water phase of the membrane (top) and in the membrane (bottom).

ρ_{H_2O} **[g cm^{-3}]**	**M$_{H_2O}$ [g mol^{-1}]**	**V$_{H_2O/SO_3}$ [L]**	λ_{V^{3+}/SO_3}	$c_{V^{3+}}$ **[mol L^{-1}]**	**K**
1	18	0.216	0.137	0.634	0.396
**weight normalization**	**EW [g mol^{-1}]**	$c_{V^{3+}}$ **[mol kg^{-1}]**	**K**	$c_{V^{3+}}$ **[mol L^{-1}]**	**K**
membrane $\lambda_{H_2O/SO_3} = 0$	1100	0.125	0.11	0.247	0.15
membrane $\lambda_{H_2O/SO_3} = 12$	1316	0.104	0.09	0.206	0.13
membrane $\lambda_{H_2O/SO_3} = 12$ including $\lambda_{V^{3+}/SO_3} = 0.137$	1323	0.104	0.09	0.205	0.13

2. Estimation of information depth in hydrated Nafion

The information depth of a $\lambda_{V^{3+}/SO_3} = 0.137$ and a $\lambda_{V^{3+}/SO_3} = 0.000137$ was calculated using the absorption coefficients from McMaster *et al.* [2] at the V Kα line of 4952 eV, with a $\lambda_{H_2O/SO_3} = 12$ and a density of the hydrated membrane of ρ_{Nafion}=1.98 g cm^{-3}. The EW of 1100 g n(SO_3)$^{-1}$ represents the Nafion fraction. The mass of the electrolyte is 223 g n(SO_3)$^{-1}$ and 216 g n(SO_3)$^{-1}$ for the two concentrations. Resulting in an electrolyte fraction of 0.17 and 0.16, respectively.

Table S2. Attenuation coefficients and weighted fractions for $\lambda_{H_2O/SO_3} = 12$, a density of the hydrated membrane of ρ_{Nafion}=1.98 g cm^{-3}, and two concentration of vanadium either $\lambda_{V^{3+}/SO_3} = 0.137$ or $\lambda_{V^{3+}/SO_3} = 0.000137$ (bottom) and the dry membrane (top).

**Elements**	**M [g mol^{-1}]**	**W**	**μ (4952 eV) [cm^2 g^{-1}]**	**W· μ [cm^2 g^{-1}]**
**Nafion**				
**$C_7HF_{13}O_5SC_2F_4$**	**544**	**1100 g n(SO_3)$^{-1}$**		
C	12	0.19	19.1	3.61
H	1	0.002	0.4	0.001
F	19	0.56	69.4	39.21
O	16	0.14	48.8	6.82
S	32	0.06	360.5	20.17
**Water**				
**$V_{0.137}$·12 H_2O**		**223 g n(SO_3)$^{-1}$**		
V	51	0.03	96.3	3.02
O	16	0.9	48.8	42.00
H	1	0.1	0.4	0.04
**$V_{0.00014}$· 12 H_2O**		**216 g n(SO_3)$^{-1}$**		
V	51	0.00003	96.3	0.003
O	16	0.9	48.8	42.00
H	1	0.1	0.4	0.04

The μ_{lin} of the high and low vanadium concentrations of the Nafion water body are obtained by the following calculation

$$\mu_{lin-high} = \left((\sum W \cdot \mu)_{Nafion} \cdot \frac{1100}{1100+223} + (\sum W \cdot \mu)_{Water} \cdot \frac{223}{1100+223} \right) \cdot \rho_{Nafion}$$

$$\mu_{lin-low} = \left((\sum W \cdot \mu)_{Nafion} \cdot \frac{1100}{1100+216} + (\sum W \cdot \mu)_{Water} \cdot \frac{216}{1100+216} \right) \cdot \rho_{Nafion}$$

The information depth is the reciprocal of the linear absorption coefficient μ_{lin}. The resulting μ_{lin} and information depth are given in the table:

Table S3. Linear attenuation coefficients μ_{lin} and information depths of $\lambda_{H_2O/SO_3} = 12$ for both vanadium concentrations of $\lambda_{V^{3+}/SO_3} = 0.137$ and a $\lambda_{V^{3+}/SO_3} = 0.000137$ in the membrane.

**Nafion condition**	**μ_{lin} (4952 eV) [cm^{-1}]**	**d_{inf} [μm]**
$\lambda_{H_2O/SO_3} = 12$ $\lambda_{V^{3+}/SO_3} = 0.137$	130.0	76.9
$\lambda_{H_2O/SO_3} = 12$ $\lambda_{V^{3+}/SO_3} = 0.000137$	129.2	77.4

The difference of the information depth between the two extreme concentrations is less than 1%. From this it can be concluded that the matrix effects are constant and the fluorescence signal scales with the vanadium concentration.

3. MD simulation for $\lambda_{H_2O/SO_3} = 2$, $\lambda_{H_2O/SO_3} = 6$ and $\lambda_{H_2O/SO_3} = 12$

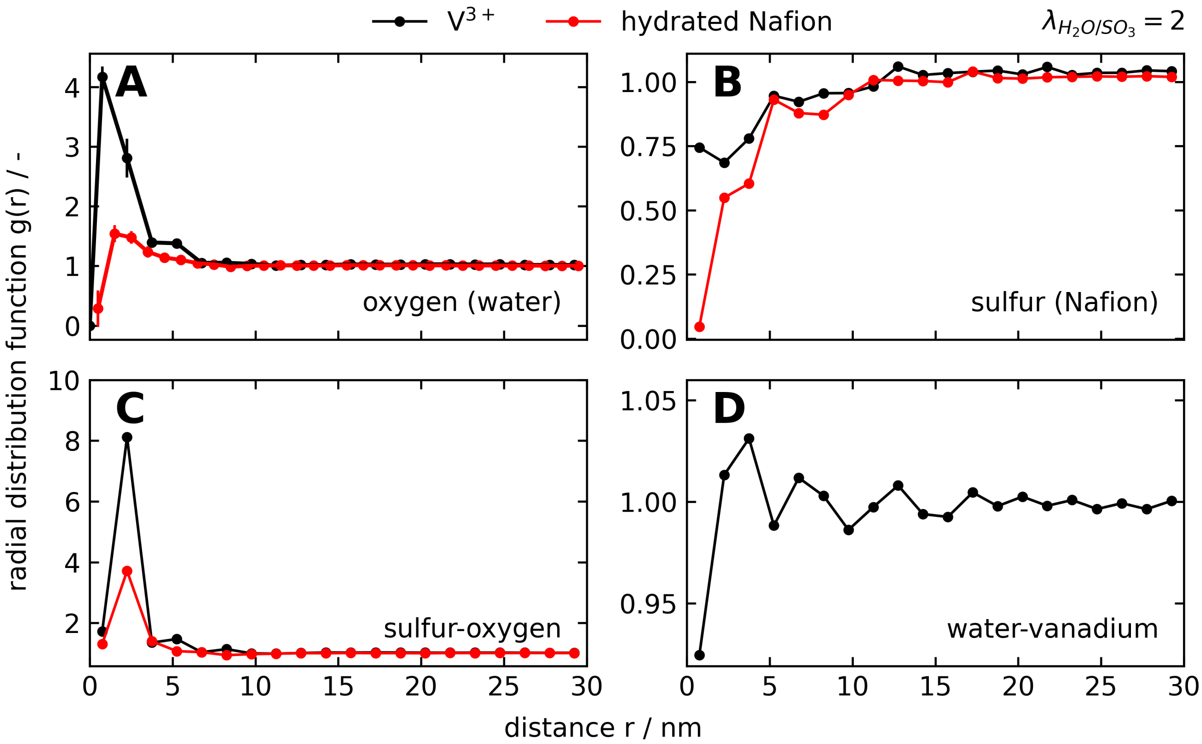

Figure S1. Simulated radial distribution function g(r) for $\lambda_{H_2O/SO_3} = 2$. (**A**) between oxygen atoms of two water molecules, (**B**) between the sulfur atoms of different Nafion molecules, (**C**) between sulfur and oxygen atoms, and (**D**) between water and vanadium atoms.

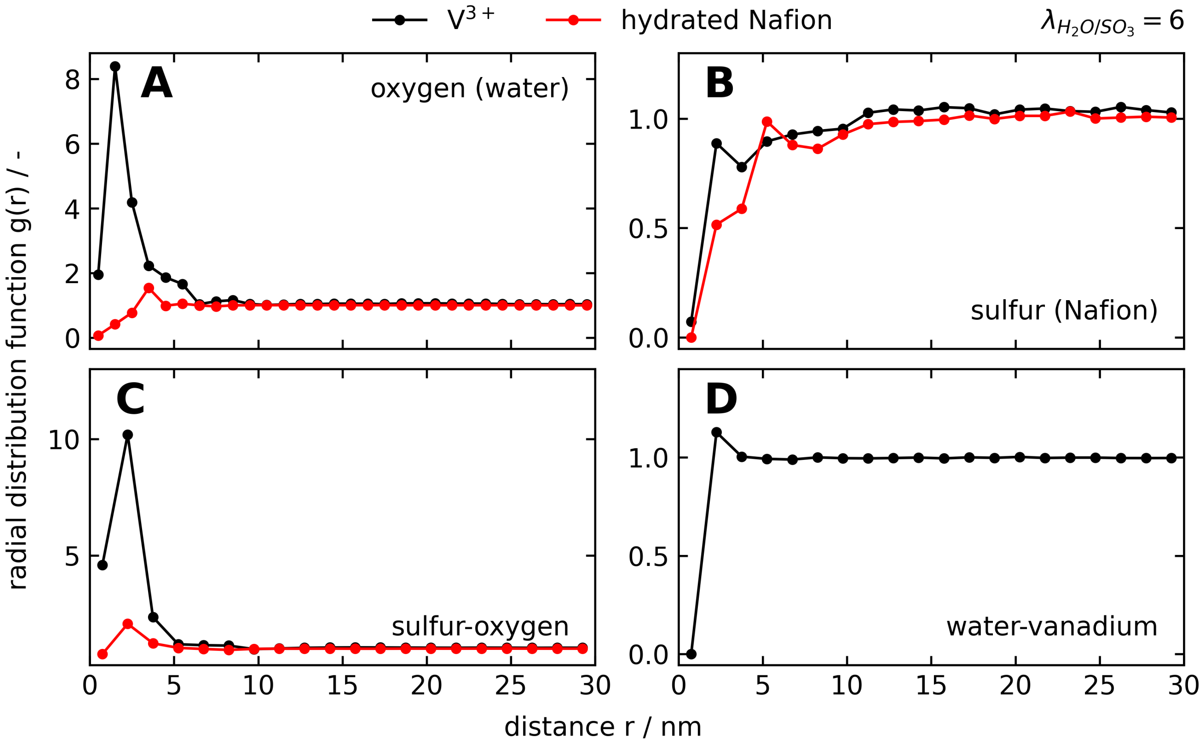

Figure S2. Simulated radial distribution function g(r) for $\lambda_{H_2O/SO_3} = 6$. (**A**) between oxygen atoms of two water molecules, (**B**) between the sulfur atoms of different Nafion molecules, (**C**) between sulfur and oxygen atoms, and (**D**) between water and vanadium atoms.

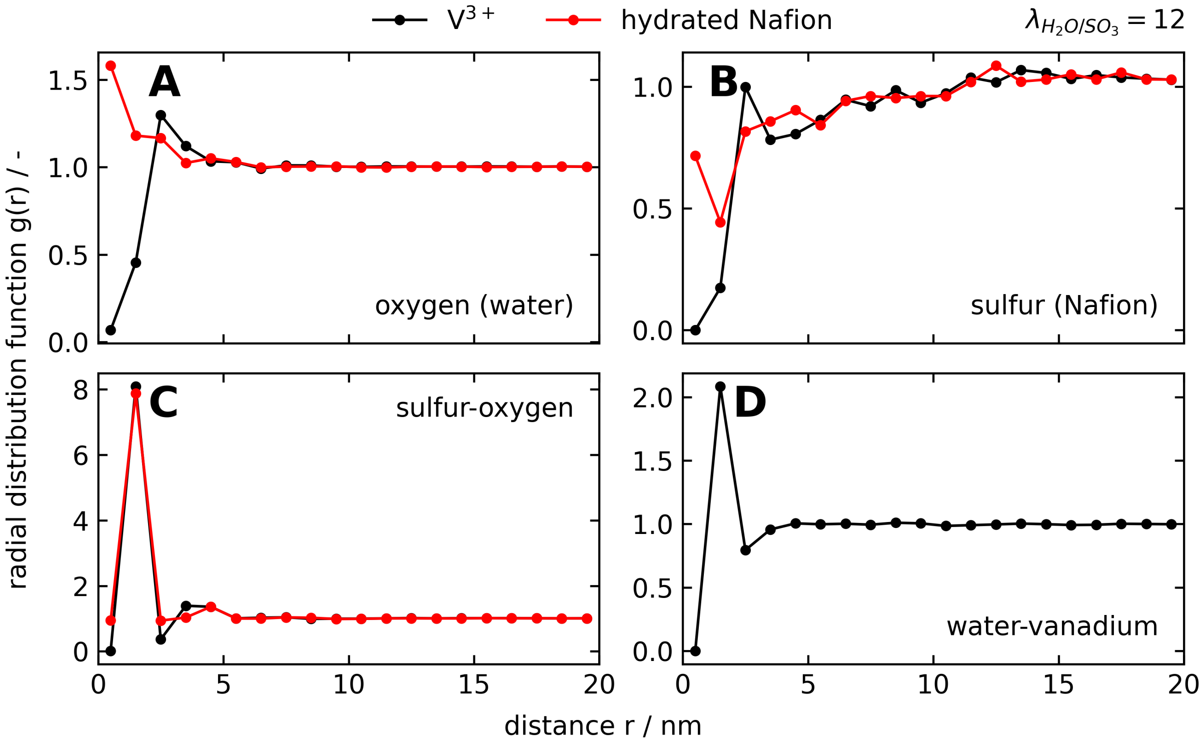

Figure S3. Simulated radial distribution function g(r) for $\lambda_{H_2O/SO_3} = 12$. (**A**) between oxygen atoms of two water molecules, (**B**) between the sulfur atoms of different Nafion molecules, (**C**) between sulfur and oxygen atoms, and (**D**) between water and vanadium atoms.

4. Determination of C_s - Derivation and errors

The intersection of two linear functions with their slopes m_i and their intercepts b_i is calculated from equating both functions and rewrite after x.

$$m_1 \cdot x + b_1 = m_2 \cdot x + b_2$$

$$m_1 \cdot x - m_2 \cdot x = b_2 - b_1$$

$$x = \frac{b_2 - b_1}{m_1 - m_2}$$

Estimating the absolute error for the intersection assumes independent errors for each linear function as well as normal distributed uncertainties. The error is derived from error propagation using standard errors σ_i from the linear fits.

$$\sigma_x^2 = \left(\frac{\partial x}{\partial b_1}\right)^2 \cdot {\sigma_{b1}}^2 + \left(\frac{\partial x}{\partial b_2}\right)^2 \cdot {\sigma_{b2}}^2 + \left(\frac{\partial x}{\partial m_1}\right)^2 \cdot {\sigma_{m1}}^2 + \left(\frac{\partial x}{\partial m_2}\right)^2 \cdot {\sigma_{m2}}^2$$

With the partial differentials inserted into the previous equation:

$$\sigma_x^2 = \left(-\frac{1}{m_1 - m_2}\right)^2 \cdot {\sigma_{b1}}^2 + \left(\frac{1}{m_1 - m_2}\right)^2 \cdot {\sigma_{b2}}^2 + \left(-\frac{b_2 - b_1}{(m_1 - m_2)^2}\right)^2 \cdot {\sigma_{m1}}^2 + \left(\frac{b_2 - b_1}{(m_1 - m_2)^2}\right)^2 \cdot {\sigma_{m2}}^2$$

By factoring out $\frac{1}{(m_1 - m_2)^2}$ it yields:

$$\sigma_x^2 = \frac{1}{(m_1 - m_2)^2} \cdot \left({\sigma_{b1}}^2 + {\sigma_{b2}}^2 + \frac{(b_2 - b_1)^2}{(m_1 - m_2)^2} \cdot {\sigma_{m1}}^2 + \frac{(b_2 - b_1)^2}{(m_1 - m_2)^2} \cdot {\sigma_{m2}}^2 \right)$$

With $m_1 - m_2 = \frac{b_2 - b_1}{x}$ this can be rewritten as:

$$\sigma_x^2 = \frac{x^2}{(b_2 - b_1)^2} \cdot \left({\sigma_{b1}}^2 + {\sigma_{b2}}^2 + \frac{(b_2 - b_1)^2}{(m_1 - m_2)^2} \cdot {\sigma_{m1}}^2 + \frac{(b_2 - b_1)^2}{(m_1 - m_2)^2} \cdot {\sigma_{m2}}^2 \right)$$

This can be simplied to:

$$\sigma_x^2 = x^2 \cdot \left(\frac{{\sigma_{b1}}^2 + {\sigma_{b2}}^2}{(b_2 - b_1)^2} + \frac{{\sigma_{m1}}^2 + {\sigma_{m2}}^2}{(m_1 - m_2)^2} \right)$$

The final equation is given with:

$$\sigma_x = \sqrt{\frac{\sigma_{b_1}^2 + \sigma_{b_2}^2}{(b_2 - b_1)^2} + \frac{\sigma_{m_1}^2 + \sigma_{m_2}^2}{(m_1 - m_2)^2}} \cdot x$$

The C_S determination starts with setting up the linear fits with proper fitting regions. Using Figure 6A linear ranges can be estimated for both fits. The first linear fit was performed from -50 μm to 2 μm and the second fit from -2 μm to 16 μm leading to a total of 2.25 mio. possible combinations. Setting up this fitting regions will definitely include the linear ranges. The fit is restricted to have at least 6 data points for good statistics. Each fit starts on the left border and ends on the right border. The opposite combination is also possible, but is excluded to remain only the unique combinations. Figure S4 shows the results from the fitting routine with standard errors and also including R^2.

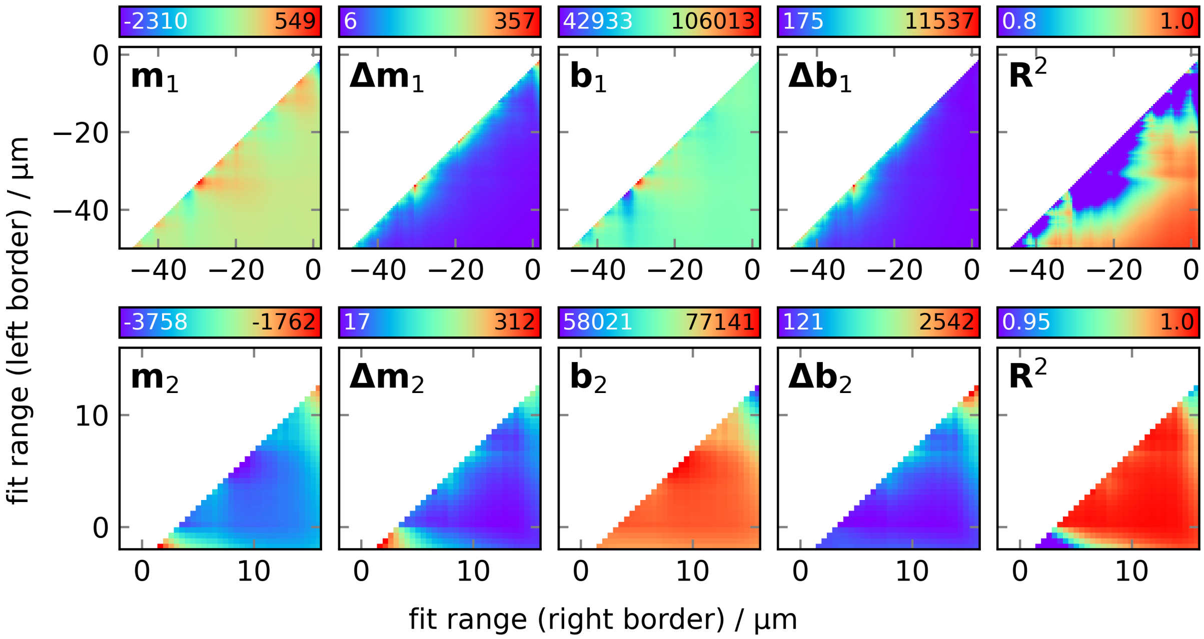

Figure S4. Fit results for two linear functions in the range from -50 μm to 2 μm and from -2 μm to 16 μm, respectively.

The linear range is indicated with low errors for the coefficients and high R^2 for the corresponding fits. The second fit range yields high R^2 even after leaving the linear range due to the limited number of data points. As all uncertainties are estimated to be

normal distributed, the interception of both functions will be the convolution of two normal distributions and can be calculated for all possible combinations (2.25 mio.). From this the interception error can also be determined.

5. Errors in Diffusion coefficient determination

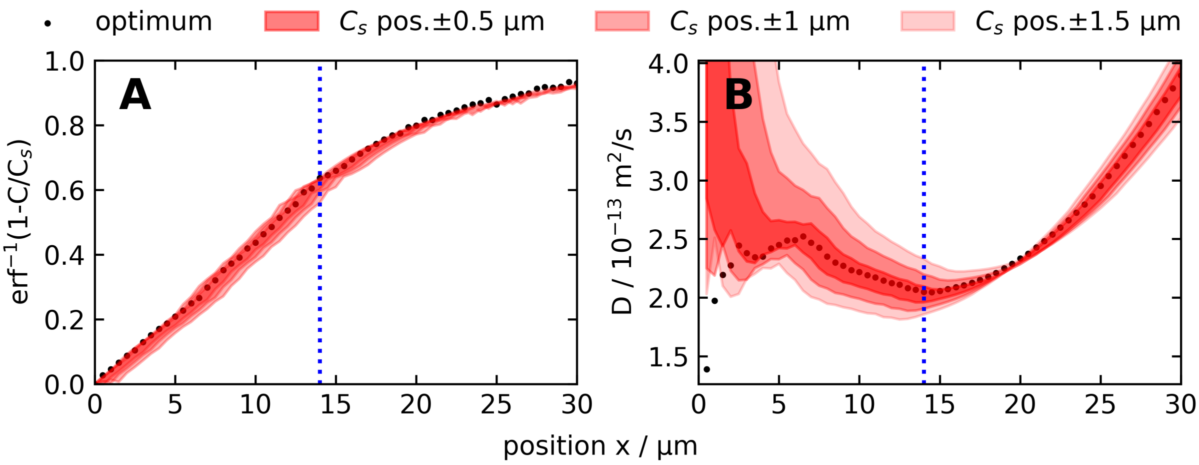

Figure S5. Influences of errors in determining the position for C_s on (**A**) the inverse error function and (**B**) subsequently on the diffusion coefficient D. The linear range for the optimal case is denoted with the blue dots in both subplots.

The resulting diffusion coefficients from Figure S5 are noted in the following table. Errors from the determination of the C_s position are given as well as errors from the fitting range on the corresponding C_s positions.

Table S4. Determined diffusion coefficients D allowing errors in position determination of C_s and fitting range.

**position error** C_S	**value**	**Diffusion coefficient** D [10^{-13} m^2s^{-1}] **position error fitting range** ± 0.5 µm	± 1.0 µm	± 1.5 µm
- 1.5 µm	2.22	2.22-2.23	2.23-2.24	2.24-2.27
- 1.0 µm	2.15	2.15	2.16-2.18	2.17-2.20
- 0.5 µm	2.07	2.07-2.08	2.09	2.10-2.11
optimum	2.05	2.05-2.07	2.06-2.08	2.07-2.11
+ 0.5 µm	1.96	1.96-1.98	1.97-1.99	1.99-2.02
+ 1.0 µm	1.89	1.89-1.90	1.90-1.91	1.92-1.94
+ 1.5 µm	1.81	1.82-1.83	1.83	1.86

References

1. Lutz, C.; Breuckmann, M.; Hampel, S.; Kreyenschmidt, M.; Ke, X.; Beuermann, S.; Schafner, K.; Turek, T.; Kunz, U.; Buzanich, A.G.; et al. Characterization of Dimeric Vanadium Uptake and Species in Nafion™ and Novel Membranes from Vanadium Redox Flow Batteries Electrolytes. *Membranes* **2021**, *11*, 576. https://doi.org/10.3390/membranes11080576.
2. McMaster, W.H.; Del Kerr Grande, N.; Mallett, J.H.; Hubbell, J.H., Eds. *Compilation of X-Ray Cross Sections: Lawrence Livermore National Laboratory Report UCRL-50174 Sec. II Rev. 1*; Lawrence Radiation Laboratory, University of California: Livermore, 1969.

Disclaimer/Publisher's Note: The statements, opinions and data contained in all publications are solely those of the individual author(s) and contributor(s) and not of MDPI and/or the editor(s). MDPI and/or the editor(s) disclaim responsibility for any injury to people or property resulting from any ideas, methods, instructions or products referred to in the content.
